# The rs3957357C>T SNP in *GSTA1* Is Associated with a Higher Risk of Occurrence of Hepatocellular Carcinoma in European Individuals

**DOI:** 10.1371/journal.pone.0167543

**Published:** 2016-12-09

**Authors:** Hanane Akhdar, Said El Shamieh, Orlando Musso, Romain Désert, Wissam Joumaa, Dominique Guyader, Caroline Aninat, Anne Corlu, Fabrice Morel

**Affiliations:** 1 Inserm, UMR991, Rennes, France; 2 Université de Rennes 1, Rennes, France; 3 Department of Medical Laboratory Technology, Faculty of Health Sciences, Beirut Arab University, Beirut, Lebanon; 4 Inserm, UMR1122, Nancy, France; 5 CHU de Rennes, Service des Maladies du Foie, Rennes, France; University of Navarra School of Medicine and Center for Applied Medical Research (CIMA), SPAIN

## Abstract

Glutathione S-transferases (GSTs) detoxify toxic molecules by conjugation with reduced glutathione and regulate cell signaling. Single nucleotide polymorphisms (SNPs) of GST genes have been suggested to affect GST functions and thus to increase the risk of human hepatocellular carcinoma (HCC). As *GSTA1* is expressed in hepatocytes and the rs3957357C>T (TT) SNP is known to downregulate GSTA1 mRNA expression, the aims of this study were: (i) to explore the relationship between the TT SNP in *GSTA1* and the occurrence of HCC; (ii) to measure *GSTA1* mRNA expression in HCCs. For that purpose, we genotyped non-tumor-tissue-derived DNA from 48 HCC patients and white-blood-cell-derived DNA from 37 healthy individuals by restriction fragment length polymorphism (RFLP). In addition, expression of *GSTA1* mRNA was assessed by real-time PCR in 18 matching pairs of HCCs and non-tumor livers. Survival analysis was performed on an annotated microarray dataset containing 247 HCC patients (GSE14520). The *GSTA1* TT genotype was more frequent in HCC than in non-HCC patients (27% versus 5%, respectively), suggesting that individuals carrying this genotype could be associated with 2-fold higher risk of developing HCCs (odds ratio = 2.1; p = 0.02). Also, we found that *GSTA1* mRNA expression was lower in HCCs than in non-tumor livers. HCCs expressing the highest *GSTA1* mRNA levels were the smallest in size (R = -0.67; p = 0.007), expressed the highest levels of liver-enriched genes such as *ALB* (albumin, R = -0.67; p = 0.007) and *COL18A1* (procollagen type XVIII, R = -0.50; p = 0.03) and showed the most favorable disease-free (OR = 0.54; p<0.001) and overall (OR = 0.56; p = 0.006) outcomes. Moreover, *GSTA1* was found within a 263-gene network involved in well-differentiated hepatocyte functions. In conclusion, HCCs are characterized by two *GSTA1* features: the TT SNP and reduced *GSTA1* gene expression in a context of hepatocyte de-differentiation.

## Introduction

Hepatocellular carcinoma (HCC) is the third cause of cancer-related death in the world [1]. Although the most efficient therapies remain surgical resection and liver transplantation [2], HCC recurrence rates remain high [2]. Many factors are involved in the pathogenesis of HCC, including chronic hepatitis B and C viral infections, alcohol abuse, genetic diseases and chronic exposure to genotoxins [3]. Case-control and cohort studies have suggested the association of single nucleotide polymorphisms (SNPs) in the glutathione S-transferase (GST) gene with an increased risk of occurrence of HCC [4, 5].

GSTs belong to the family of intracellular isoenzymes that mediate the conjugation of reduced glutathione to exogenous or endogenous compounds. Thus, oxidative stress products, prostaglandins, chemical carcinogens and therapeutic drugs are detoxified by GSTs [6]. The nucleophilic attack of reduced glutathione on electrophilic substrates, catalyzed by GST enzymes, represents a defense mechanism in the cell. Indeed, glutathione conjugation reduces the toxic effects of strongly reactive products on proteins and DNA [6]. Therefore, one of the roles of GSTs is to protect DNA against oxidative damage, which may lead to mutations, and in consequence, favor carcinogenesis [6]. More recently, it has been shown that GSTs also play important roles in regulating signaling pathways in a catalytic-independent manner through direct interaction with kinases, such as *c-jun* N-terminal kinase (JNK) and apoptosis signal-regulating kinase 1 (ASK1) to modulate their phosphorylation activities [7].

Eight classes of cytosolic GST are recognized in mammalian species, named *Alpha*, *Mu*, *Pi*, *Sigma*, *Theta*, *Kappa*, *Omega*, and *Zeta* [6]. Many studies showed the involvement of *GSTM1* and *GSTT1* in human carcinogenesis [6]. *GSTA1* encodes a GST belonging to the alpha class [8]. GST alpha class genes are located in chromosome 6, and constitute the most abundantly expressed GSTs in the liver [8]. The GST alpha family exhibits an important glutathione peroxidase activity that protects the cell from reactive oxygen species. In addition, they metabolize bilirubin and many anti-cancer drugs in the liver [9]. Several SNPs in *GSTs* have been shown to produce significant alterations in the metabolism of many carcinogens and chemotherapeutic agents [10] and to increase the risk of cancer (mainly oral, skin, lung, head and neck) [11–15]. Over the past few years, major efforts have been devoted to explore the relationships between *GSTT1*, *GSTM1* families and the risk of developing HCC, leading to the demonstration that *GSTT1* and *GSTM1* null genotypes may slightly increase the risk of HCC [4, 16, 17].

*GSTA1* is a relatively small gene (around 11Kb) harboring 7 SNPs with allele frequency of more than 5% in the general European population. Although the rs3957357C>T in *GSTA1* was reported to be a functional SNP affecting the transcriptional activity of its gene in the liver [17, 18], the possible implication of *GSTA1* [4, 17, 18] in HCC was not studied. Therefore, the goals of our study were: (i) to investigate the relationships between the SNP rs3957357C>T in *GSTA1* and the risk of HCC occurrence among European individuals; (ii) to measure *GSTA1* mRNA expression in HCCs and (iii) to interpret these findings in the light of patient outcome after HCC resection. We found that individuals homozygous for the TT genotype of *GSTA1* were associated with a 2-fold increase in the risk of developing HCCs and that low *GSTA1* gene expression occurs in poorly-differentiated tumors showing a bad clinical outcome.

## Patients, Materials and Methods

### 1. Genotyping assay

#### Patients and white blood cell samples for genotyping

*GSTA1* genotyping was performed on non-tumor-tissue-derived DNA from 48 HCC patients and on white-blood-cell-derived DNA from 37 healthy individuals. Liver and white blood cell samples were obtained at Rennes University Hospital between January 1999 and December 2002. The 48 patients included in this study had histopathologically confirmed HCC. Genomic DNA was extracted with the DNeasy Kit (Qiagen) according to the manufacturer’s instructions. Quality control and quantification of extracted DNA was performed by spectrophotometry (Nanodrop, ThermoFisher Scientific) and by agarose electrophoresis to check for DNA integrity.

#### Genotyping assay of rs3957357C>T *in GSTA1*

Restriction fragment length polymorphism (RFLP) genotyping of the SNP rs3957357C>T in *GSTA1* was done by amplifying a 480 base pair stretch within the promoter followed by *EarI* restriction enzyme digestion (New England Biolabs, Beverly, Massachusetts, USA). The primers used were 5’- GATCTAGGGATTTCTATATGGACCT-3’ (forward) and 5’- GTTAAACGCTGTCACCGTCCT-3’ (reverse). All PCR reactions contained 1U of Taq DNA polymerase (Roche Diagnostic, Basel, Switzerland) with 1X Buffer, 200 ng of genomic DNA, 1.5 mM MgCl_2_, 100 μM dNTPs, 0.5 μM of each primer in a final volume of 50 μl. DNA was first denatured at 95°C for 5 min, followed by 35 cycles of denaturing at 95°C for 30 sec. Annealing was done at 62°C for 1 min and extension at 72°C for 30 sec. A final extension step was also performed at 72°C for 7 min. The genotyping analysis of the 480 base-pair amplicon was performed through digestion with *EarI* restriction enzyme at 37°C for 2 hr.

### 2. Gene expression analysis

#### Patients and tissue samples

Liver tissues were obtained from 36 patients at Bordeaux University Hospital in France, between May 1991 and December 1997, as described [19]. The microscopic features of tumors diagnosed as HCC were reviewed and annotated by a senior pathologist. Samples consisted of 18 HCCs and 18 matching non-tumor areas. Upon gross anatomic pathology analysis of liver samples, tumor size was defined as the largest diameter of the tumor (cm) or the diameter of the largest tumor (when multiple HCCs were present). Complying with the French Bioethics law at the time of patient inclusion in this study, the participants provided verbal informed consent to their respective surgeons. Participant consent was recorded in medical files. The study protocol complied with French laws and regulations and was approved by INSERM’s Institutional Review Board (number 01–036) in the context of the National Network of Liver Biological Resource Centers. Paraffin-embedded tissue blocks were processed for histology (H&E-Saffran and Sirius red staining), and then classified using standard systems [20, 21]. Fresh mirror image tissue fragments, adjacent to the paraffin-embedded tissue blocks, were snap frozen at -80°C in N_2_-cooled isopentane and stored at -80°C under quality-controlled conditions [22, 23]. White blood cell samples for genotyping were not available for these patients.

#### RNA extraction and real-time PCR analysis of mRNA expression

Total RNA was extracted as described, using the cesium chloride ultracentrifugation method [19]. Using a High-Capacity cDNA Archive kit (Applied Biosystems, Foster City, CA), total mRNA was reverse-transcribed into cDNA. Real-time PCR was performed with the SYBR Green PCR Master Mix (Applied Biosystems) and the ABI prism 7300 PCR station (Applied Biosystems). Primers were previously described [22, 23]. The amplification curves were read with the 7300 SDS software using the comparative cycle threshold method. All experiments were carried out in triplicates in a total reaction volume of 20 μl containing 0.5 μM of each specific primer. Negative and internal controls were included. The relative quantification of *GSTA1* mRNA levels was calculated after normalization to *18S* RNA as the housekeeping gene. A melting curve analysis was then conducted to verify amplification specificity. The expression levels of the 18S housekeeping gene were used for normalization. RNA expression values were calculated by the ΔΔCt method and normalized by *[(LNi-mean*_*LNi→n*_*)/SD*_*LNi→n*_*]*, where *LN*, natural logarithm; *i→n*, from the first to the last value.

### 3. Linkage disequilibrium analysis using HapMap data

#### Patients

We based our analyses on the Centre d'Etude du Polymorphisme Humain (CEPH) population. It is a population composed of 30 family trios and genotyped by the International HapMap Consortium [24] downloaded from the HapMap Web site (http://www.hapmap.org; HapMap Data Phase III/Rel#2).

#### SNP selection and linkage disequilibrium analysis

We selected the gene regions of *GSTA1*. Pedigree information and genotypes for the CEPH cohort were downloaded from the HapMap homepage and were further analyzed using Haploview version 3.2 [25].

### 4. Statistical analyses

Statistical analyses were performed with the SPSS® statistical software version 20 (SPSS, Inc, Chicago, Illinois) and R (version 3.3.0). Statistical significance of the differences between means was calculated by the Mann-Whitney U test. Significance of monotonic non-linear relationships between variables was assessed by Spearman’s rank order correlation tests. Survival analyses were performed with the Log Rank test and Kaplan-Meier curves. A *χ*^*2*^ test was performed to determine whether genotype frequencies were in Hardy-Weinberg equilibrium. Logistic regression models were further used to assess the association between rs3957357C>T in *GSTA1* and the status of HCC after adjustment for age and gender. The significance level was set at p<0.05. Gene co-expression networks were generated and visualized as we previously described [22, 23] by Weighted Correlation Network Analysis (WGCNA, WGCNA package) [26] and visually integrated with Cytoscape [27], with correlation coefficient thresholds > 0.40 (positive correlations) or <– 0.40 (negative correlations).

## Results

### SNP rs3957357C>T in *GSTA1* is associated with higher risk of HCC

**Fig 1:**
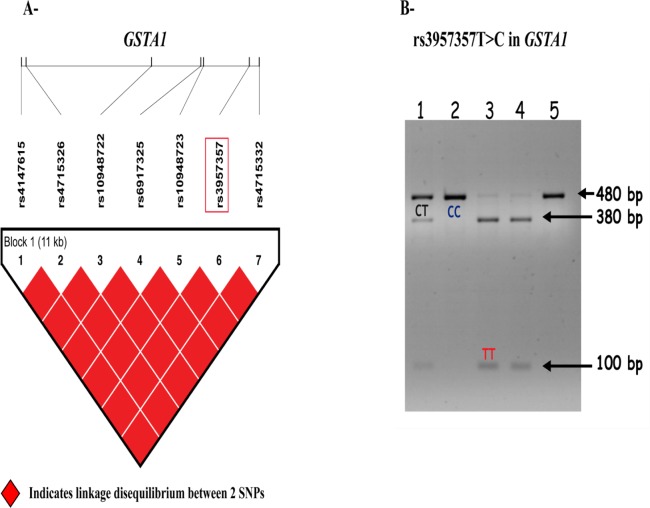
Genetics and bio-informatics analysis of rs3957357C>T in *GSTA1*. A, Linkage disequilibrium analysis between *GSTA1* single nucleotide polymorphisms in the CEPH-HapMap population visualized with the Haploview software. Linkage disequilibrium data for *GSTA1* gene shows 7 SNPs, among them rs3957357 (highlighted in red). All the single nucleotide polymorphisms are in high linkage disequilibrium. Red squares indicate complete linkage disequilibrium between two single nucleotide polymorphisms. SNPs: single nucleotide polymorphisms. B, Representative image of a PCR-RFLP experiment. The presence of the T allele in rs3957357C>T of *GSTA1* gene leads to the production of 380 bp and 100 bp fragments, while the amplicons carrying the C allele remain undigested.

Analysis of the HapMap data showed that the 7 SNPs known in *GSTA1* were in Linkage disequilibrium (LD) in European individuals (Fig 1A). This observation implies that genotyping of one SNP is sufficient to infer the genotypes of the remaining ones. In other words, if one SNP has the minor allele, all the others in LD will probably have also the minor allele. Analysis of the SNP rs3957357C>T by PCR-RFLP revealed that the T allele led to the production of 380 bp and 100 bp fragments, while amplicons containing the C allele remained undigested (Fig 1B).

**Fig 2:**
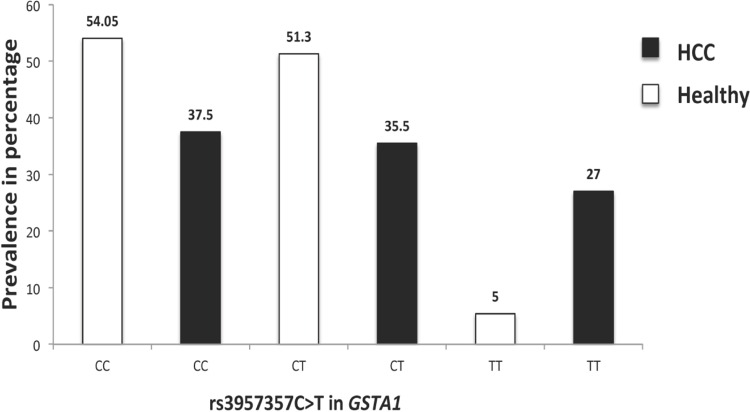
Prevalence of healthy individuals and patients with hepatocellular carcinoma according to the rs3957357C>T genotype in the *GSTA1* gene. Stratification according to the rs3957357C>T genotype in *GSTA1* shows a 5-fold increase in the prevalence of individuals with HCC carrying the TT genotype (27%), compared with healthy individuals and carrying the same genotype (5%).

We first verified that genotypes and allele frequencies in 37 healthy and 48 individuals with HCC were consistent with the Hardy-Weinberg equilibrium by a chi-square *(χ*^*2*^*)* test (not shown). Then, in order to investigate the relationships between *GSTA1* SNPs and HCC, we compared the frequencies of the three genotypes of rs3957357C>T of *GSTA1* in these two groups of individuals. We found that the TT genotype was more frequent in HCC patients (27%) than in healthy individuals (5%) (Fig 2). Furthermore, using a logistic regression model, we found that individuals homozygous for the TT genotype had a 2-fold increase in the risk of developing HCC (odds ratio = 2.1; p = 0.02).

### Low *GSTA1* expression in HCC is associated with bad outcome

**Fig 3:**
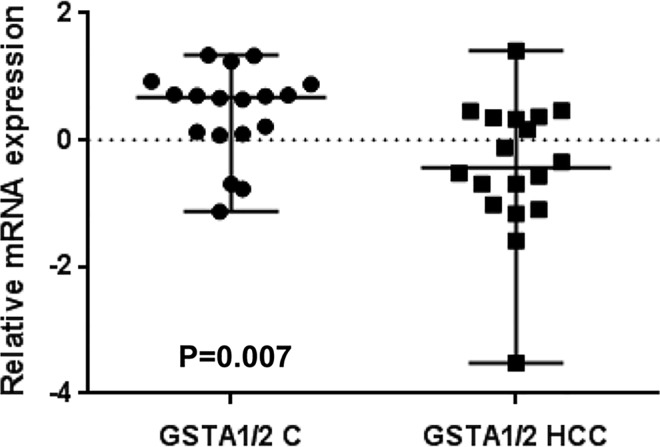
*GSTA1* expression in hepatocellular carcinomas and matching non-tumor livers. Real-time PCR analysis of normalized *GSTA1* mRNA expression in 36 frozen liver samples including (HCCs), and matching non-tumor livers (C). RNA expression values were calculated by the ΔΔCt method. Statistical significance of the differences between means was assessed by the Mann-Whitney “U” test (p = 0.007).

**Fig 4:**
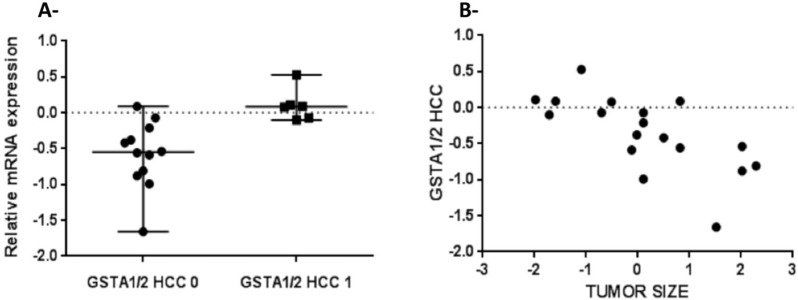
*GSTA1* mRNA levels are negatively correlated with tumor size in HCCs. A, Real-time PCR analysis of normalized *GSTA1* mRNA expression in 18 HCCs. HCC 0 (large HCC), HCC1 (Small HCC, <3 cm) (p = 0.002). B, Real-time PCR analysis of *GSTA1* mRNA expression in 18 HCCs and tumor size are negatively correlated (Spearman’s R = -0.67; p = 0.007).

**Fig 5:**
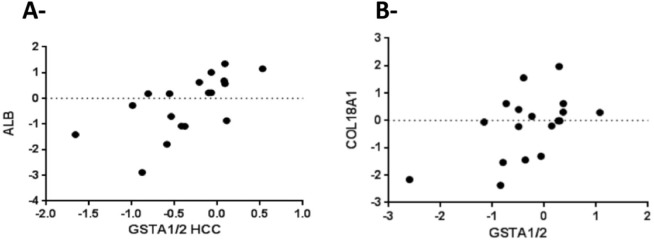
Correlation analysis of *GSTA1*, albumin (ALB) and procollagen type XVIII (COL18A1) mRNA expression in hepatocellular carcinomas. A, Real-time PCR analysis of albumin mRNA expression levels in 18 HCCs (Spearman’s R = 0.67; p = 0.001). B, Real-time PCR analysis of procollagen type XVIII mRNA expression levels in 18 HCCs (Spearman’s R = 0.50; p = 0.03).

**Fig 6:**
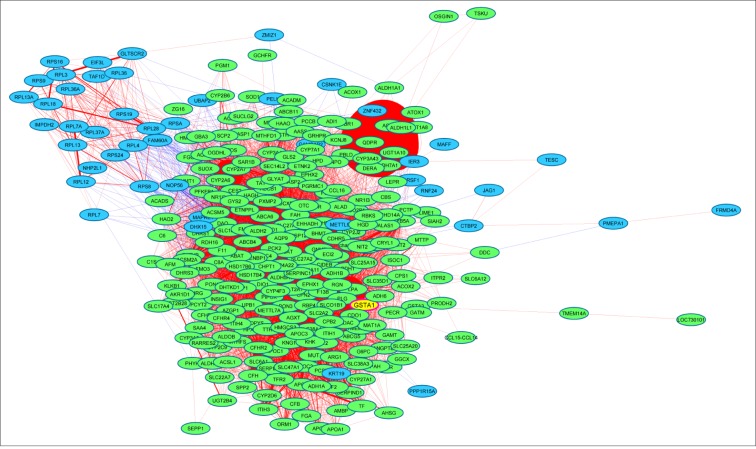
*GSTA1* co-expression network in 247 human HCCs. *GSTA1* is highlighted as a yellow node. Weighted correlation network analyses of mRNA expression data was visualized with Cytoscape with correlation coefficient thresholds > 0.40 (positive correlations) or <– 0.40 (negative correlations). Closeness between nodes is proportional to the number of connections. Thickness of links is proportional to the correlation coefficients between genes. Green nodes, positively correlated genes; blue nodes, negatively correlated genes; red links, positive correlation; blue links, negative correlation.

Because the SNP rs3957357C>T is known to reduce *GSTA1* transcriptional activity, we analyzed *GSTA1* mRNA expression by real-time PCR in 18 matching pairs of tumor and non-tumor liver tissues from patients undergoing resection of HCCs. *GSTA1* mRNA expression was higher in non-tumor livers than in HCCs (P = 0.007, Fig 3) and also higher in small than in large HCCs (P = 0.002, Fig 4A). Consistently with these data, *GSTA1* mRNA levels were negatively correlated with tumor size (P = 0.007, Fig 4B), and positively correlated with the mRNA expression of the liver-enriched genes albumin (P = 0.001, Fig 5A) and procollagen type XVIII (P = 0.03, Fig 5B). Then, we explored an external microarray-based mRNA expression dataset from 247 HCC patients [28] (GSE14520), which confirmed positive correlations of *GSTA1* with albumin and procollagen type XVIII (not shown) and showed additional positive correlations with the liver-enriched transcription factor HNF4A [29] and the liver-specific methionine adenosyltransferase MAT1A [30] (S1 Table). These data led us to construct a *GSTA1* co-expression network in the 247-HCC GSE14520 dataset applying a high-stringency correlation coefficient threshold (Pearson’s correlation coefficient > 0.40 or < –0.40). Weighted correlation network analyses (WGCNA) [26] revealed a 263 gene set positively correlated and a 57 gene set negatively correlated with *GSTA1* in human HCCs. Positively and negatively correlated genes formed two tightly co-expressed gene clusters (Fig 6). Positively-correlated genes were involved in well-differentiated hepatocyte cell functions, such as fibrinolysis, amino-acid catabolism, urea metabolism, gluconeogenesis and drug metabolism. In this cluster, we also identified that under-represented cell functions were those associated with cell proliferation and cell cycle regulation (S2 Table). Negatively-correlated genes were mainly associated with protein synthesis (S3 Table). Of note, this cluster contained KRT19 and JAG1 (Fig 6). Both genes are markers of cholangiocyte lineage commitment of liver progenitor cells and KRT19 is associated with bad outcome in human HCCs [31]; JAG1 is a ligand of Notch family receptors, a pathway that is active upon cholangiocyte commitment [32].

**Fig 7:**
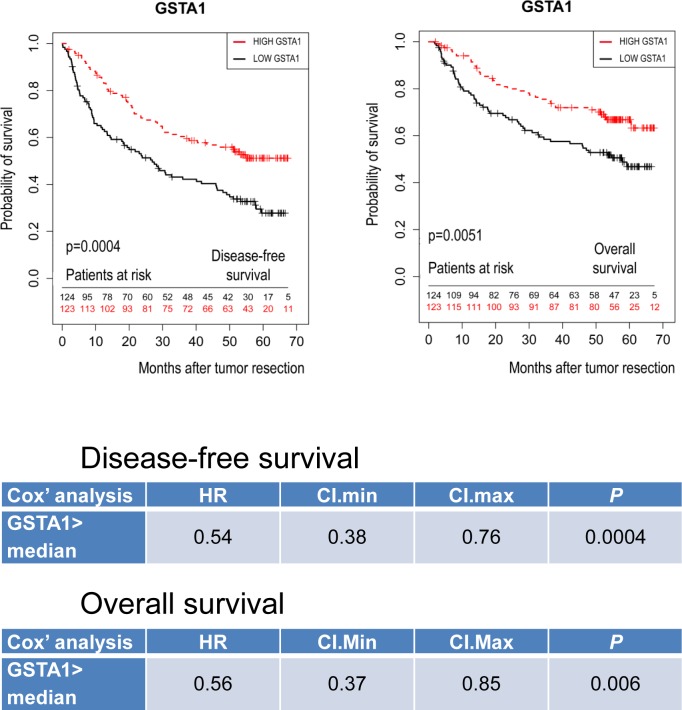
Loss of *GSTA1* mRNA expression is associated with bad outcome after curative HCC resection. *GSTA1* mRNA expression was significantly related to disease-free and overall survival by Log-Rank and Cox regression analysis on an annotated microarray dataset from 247 HCC-resected patients (Gene Expression Omnibus, GSE14520. Data were normalized and filtered as described).

Since preserved *GSTA1* expression in HCCs was associated with well-differentiated hepatocyte functions and HCCs retaining a well-differentiated hepatocyte-like phenotype show a relatively favorable outcome [3], we further analyzed the microarray dataset GSE14520, which included patient outcome annotations [28]. Kaplan-Meier curves, log-rank and Cox analyses consistently showed that a low *GSTA1* expression in HCCs was associated with unfavorable disease-free and overall outcome (P = 0.0004 and P = 0.0006 respectively, Fig 7).

## Discussion

The genetics of drug-metabolizing enzymes such as cytochromes P450 and GSTs influence individual susceptibility to cancer [33]. In particular, it has been hypothesized that overexpression of GSTs could result in faster detoxification of anti-tumor drugs and promote resistance [33]. However, we show here that this working model does not apply to all GSTs. Actually, *GSTA1* gene expression decreases in HCCs with respect to non-tumor livers. Moreover, low *GSTA1* expression in tumors correlates with indicators of unfavorable clinical outcome.

We found that individuals homozygous for the TT genotype have a 2-fold increase in the risk of developing HCC. Along these lines, rs3957357T in *GSTA1* was reported to be associated with a higher risk of developing several malignant diseases, such as colorectal [34], and breast cancer [35]. Noteworthy, rs3957357T in *GSTA1* was shown to be associated with a reduced transcriptional activity [8], resulting in a low hepatic expression of *GSTA1* [8]. Here, we showed that *GSTA1* mRNA expression was down-regulated in HCCs with respect to non-tumor tissues. Importantly, low *GSTA1* expression in HCCs was proportional to high tumor size and low expression of the liver-enriched genes albumin [36] and procollagen type XVIII [37]. In addition, *GSTA1* expression, in an independent set of 247 HCCs, was associated with the expression of liver-enriched genes, such as HNF4A, MAT1A, SDS, ARG1 as well as of several members of the cytochrome P450 family involved in well-differentiated hepatocyte-specific metabolic functions. Moreover, in an independent meta-analysis of 603 HCCs, *GSTA2* (a paralog of *GSTA1)* was predominantly expressed by a subclass of well-differentiated HCCs [38]. Furthermore, decreased *GSTA1* mRNA expression in 247 HCC patients was associated with an increased risk of tumor recurrence and bad overall outcome after tumor resection. Of note, large HCCs composed of poorly-differentiated tumor cells, which express low levels of hepatocyte-enriched genes such as procollagen type XVIII, are very aggressive tumors, frequently showing p53 mutations, genomic instability, stem cell features, drug resistance, high proliferation and reduced survival [38]. Therefore, it is reasonable to ask whether *GSTA1* functions as a tumor suppressor. One of the limitations of this study was that HCC expression of *GSTA1* and genotyping for the TT SNP were performed in different patient cohorts, which precludes the search for a causal relationship between the SNP and clinical outcome.

Tumor heterogeneity in human hepatocellular carcinomas is a major limitation to the development of efficient therapeutic strategies [2]. However, a common theme in the malignant progression of these tumors is hepatocyte de-differentiation, whereby tumor cells lose the hepatocyte-specific phenotype. These cells proliferate autonomously and acquire the ability to invade and destroy normal tissues [22, 23, 39]. In conclusion, our data suggest that the TT genotype of *GSTA1* is associated with an increased risk of occurrence of hepatocellular carcinomas and that decreased expression of *GSTA1* is a marker of advanced and highly aggressive hepatocellular carcinomas.

## Supporting Information

S1 Table Pearson’s correlation coefficients between the mRNA expression levels of the indicated genes in 247 HCCs from GSE14520 microarray dataset. Statistically significant correlations are highlighted in red. ***, p < 0.001.
(XLSX)

S2 Table Over-represented *(rows number 2 to 353)* and under-represented *(rows number 355 to 402)* gene ontology attributes in the 263 gene set positively correlated with *GSTA1* (Pearson’s correlation coefficient > 0.4) in 247 human HCCs from the GSE14520 microarray dataset. Gene ontology enrichment analysis was performed with FuncAssociate 2.0 [40] using *hgnc_symbol* as namespace and p<0.05 as significance cutoff. *LOD* is the log_10_ of the odds value indicating over- or under-representation.
(XLSX)

S3 Table Over-represented gene ontology attributes in the 57 gene set negatively correlated with *GSTA1* (Pearson’s correlation coefficient < –0.4) in 247 human HCCs from the GSE14520 microarray dataset. Gene ontology enrichment analysis was performed with FuncAssociate 2.0 [40] using *hgnc_symbol* as namespace and p<0.05 as significance cutoff. *LOD* is the log_10_ of the odds value indicating overrepresentation. No under-represented gene ontology attributes were identified in this gene set.
(XLSX)
